# Basilar artery tortuosity as a predictive factor for the efficacy of heparin adjuvant therapy in unilateral idiopathic sudden sensorineural hearing loss

**DOI:** 10.1016/j.bjorl.2020.06.017

**Published:** 2020-08-06

**Authors:** Woongsang Sunwoo

**Affiliations:** a Gachon University Gil Medical Center, Department of Otorhinolaryngology-Head and Neck Surgery, Incheon, South Korea; b Gachon University, College of Medicine, Department of Otorhinolaryngology-Head and Neck Surgery, Incheon, South Korea

**Keywords:** Hearing loss, unilateral, Magnetic resonance imaging, Basilar artery, Ischemia, Heparin

## Abstract

**Introduction:**

Cochlear ischemia is hypothesized as one of the major etiologies of idiopathic sudden sensorineural hearing loss. Therefore, anticoagulant therapies are designed to be beneficial in certain patients with this condition.

**Objective:**

This study aimed to determine which patients with idiopathic sudden sensorineural hearing loss would benefit from heparin treatment as adjuvant therapy.

**Methods:**

In total, 134 patients who underwent magnetic resonance imaging for unilateral idiopathic sudden sensorineural hearing loss at a tertiary referral hospital between January 2014 and December 2018 were included in this retrospective study. All patients received Intratympanic steroid injections or heparin therapy plus oral corticosteroids. Radiological parameters of the vertebrobasilar system and clinical data from pre- and post-treatment assessments were analyzed.

**Results:**

Most patients (71.6%) had a tortuous basilar artery The 65 patients with severe-to-profound idiopathic sudden sensorineural hearing loss showed a significant relationship between idiopathic sudden sensorineural hearing loss laterality and basilar artery displacement to the opposite side (*p* = 0.036), while the 69 patients with mild-to-moderate idiopathic sudden sensorineural hearing loss did not (*p* = 0.950). Additionally, the degree of basilar artery tortuosity was significantly associated with the degree of hearing impairment in the severe-to-profound idiopathic sudden sensorineural hearing loss group (*p* = 0.015). When idiopathic sudden sensorineural hearing loss occurred on the opposite side to basilar artery displacement, the improvement of hearing was significantly greater in patients treated with heparin than in those treated with intratympanic steroids (*p* =  0.041).

**Conclusion:**

In a subset of patients with severe-to-profound idiopathic sudden sensorineural hearing loss, basilar artery tortuosity had a significant directional relationship with idiopathic sudden sensorineural hearing loss laterality. In these selected patients, a significant effect of heparin therapy on improving hearing was observed.

## Introduction

Cochlear ischemia caused by atherothrombotic events is hypothesized to be one of the major etiologies of Idiopathic Sudden Sensorineural Hearing Loss (ISSNHL).^1^ Although many studies have reported a positive association between the risk factors for ischemic vascular disease and ISSNHL,^2^, ^3^ the effect of these thromboembolic risk factors on the likelihood of developing ISSNHL is still controversial.^4^ Discrepancies in the results of previous studies suggest that several different mechanisms of pathogenesis, including viral infection and autoimmunity, can cause ISSNHL. Therefore, ISSNHL of vascular origin needs to be differentiated from other causes to enable the selection of appropriate treatments such as anticoagulant therapies.

Blood to the cochlea is supplied by the internal auditory artery, which usually arises from a branch of the Basilar Artery (BA). Because the BA is formed by the union of two Vertebral Arteries (VA) that are commonly asymmetric in size, tortuosity of the BA is frequently observed. Morphologic and hemodynamic changes in the tortuous BA can contribute to the development of atherothrombosis on the side contralateral to its angulation.^5^ Thus, BA tortuosity on the opposite side to that of hearing loss could support the inference of vascular involvement in ISSNHL.^6^ In addition, Magnetic Resonance Imaging (MRI), which is increasingly being used as a non-invasive imaging method for the examination of the vertebrobasilar system, has improved our ability to assess the vasculature when evaluating ISSNHL.^7^

Oral corticosteroids are currently widely used as the standard treatment for ISSNHL. Although there are many observational studies that suggest a treatment benefit of corticosteroid treatment, additional therapies are often required due to the lack of effectiveness of steroids based on randomized controlled trials.^8^ Since different types of treatment entail different risks, the guideline update group of the American Academy of Otolaryngology–Head and Neck Surgery Foundation made strong negative recommendation for the routine use of additional pharmacologic agents to avoid adverse events of unnecessary treatment.^9^ Theoretically, anticoagulant therapy in patients with vascular ISSNHL may be valuable to aid the recovery of hearing. Multiple studies have shown a benefit of heparin usage in some patients with ISSNHL.^10^, ^11^, ^12^, ^13^, ^14^, ^15^ The aim of this study was therefore to determine which patients would benefit from heparin treatment as adjuvant therapy for ISSNHL, and to discourage clinicians from routinely using unnecessary treatment.

## Methods

### Ethical consideration

The study protocol and a waiver of consent for retrospective chart review were approved by the Institutional Review Board of the Clinical Research Institute (GBIRB2019-419). The study was carried out in accordance with the Declaration of Helsinki.

### Subjects

This was a retrospective cohort study performed in a tertiary referral center. Electronic medical records were reviewed for the period between January 2014 and December 2018, and adult patients (> 18 years old) who were diagnosed with unilateral ISSNHL and underwent temporal bone MRI were considered for inclusion. Patients who had any identifiable cause of hearing loss were excluded. A total of 134 patients were finally included in this study.

### Interventions

All of the patients were admitted and started on treatment with a standard oral steroid protocol (a course of 48 mg methylprednisolone tapered over 11 days) less than 7 days after the onset of hearing loss. In addition to systemic steroid therapy, patients received either heparin (10,000 IU of unfractionated heparin, intravenously, daily for 5 − 10 days) or intratympanic steroid injection (ITS, five doses over 10 days of 5 mg/mL dexamethasone) as adjuvant therapy. Patients who had hypertension, diabetes, or cardiovascular disease were also given specific treatment for these conditions along with the oral steroid and adjuvant therapy.

### Audiological assessment

Hearing was evaluated using pure tone audiometry at the initial visit and at the three-month followup. The Pure Tone Average (PTA) was calculated as the arithmetic mean of the hearing thresholds (dB) at 0.5, 1, 2, and 4 kHz in the affected ear.^16^ The degree of hearing loss was classified into one of two groups using a cut-off value of 70 dB: mild-to-moderate (PTA < 70 dB) and severe-to-profound hearing loss (PTA ≥ 70 dB). The final PTA values at the three-month followup audiogram were used to assess the outcome of therapy. Based on Siegel’s criteria, the hearing recovery outcomes were classified into 4 groups: complete recovery (final PTA < 25 dB), partial recovery (> 15 dB gain and final PTA 25 − 45 dB), slight improvement (> 15 dB gain and final PTA > 45 dB), and no improvement (<15 dB gain or final PTA > 75 dB).^17^

### MRI analysis

**Figure 1:**
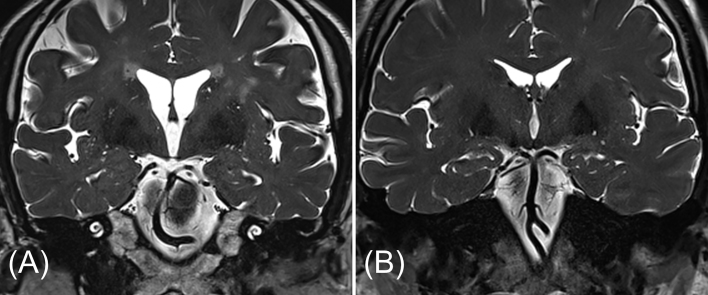
Assessment of the tortuosity of the basilar artery on coronal T2-weighted images. A, Tortuous basilar artery with marked displacement to the right. B, Straight basilar artery in the midline.

Temporal bone MRI was performed using a 3.0 T scanner (Skyra, Siemens Medical System, Erlangen, Germany). Morphologic characteristics of the vertebrobasilar system were analyzed blinded to the clinical information. Images were analyzed to determine the tortuosity (side and degree) and the diameter of the BA, and the presence of a dominant VA. The transverse diameter of the BA at the midpontine level was measured on axial T2-weighted images.^18^ BA tortuosity was determined from coronal T2-weighted images (Fig. 1). The degree of BA laterality was graded from 0 to 3 on the basis of severity (0, midline; 1, questionably off midline; 2, definite displacement; 3, reaching the cerebellopontine angle).^7^

### Statistical analysis

All statistical analyses were performed using SPSS software (version 19.0, IBM Corp., Armonk, NY, USA). Descriptive data are reported as counts (proportion, %) or mean ± Standard Deviation (SD), as appropriate. The chi square test and Fisher’s exact test were used to compare categorical variables, as appropriate for the data. For continuous variables with a normal distribution, Student's *t*-tests were used. For skewed variables, groups were compared using the Mann-Whitney *U* test. The relationship between the side affected by hearing loss and the radiological characteristics of the vertebrobasilar system was assessed using the linear-by-linear association test; *p*-values of < 0.05 were considered statistically significant.

## Results

### Demographics and clinical characteristics

We included a total of 134 patients (aged 52.2 ± 11.9 years) divided in two groups: those with mild-to-moderate hearing loss (n = 69) and those with severe-to-profound hearing loss (n = 65). The mean initial PTA in the affected ears was 52.2 ± 12.8 dB for the mild-to-moderate group and 92.9 ± 16.3 dB for the severe-to-profound group. Table 1 shows a comparison of the demographic and clinical characteristics of the two groups. There were no significant differences between the two groups with respect to age, sex, vascular risk factors, the affected side, and the time between onset and the initiation of treatment. However, there was a statistically significant difference between the groups in the presence of vertigo and the final PTA after treatment (*p* =  0.010 and < 0.001, respectively). The rates of vertigo at presentation and the follow-up PTA were higher in patients with severe-to-profound hearing loss.

**Table 1:** Demographics and clinical characteristics of the patients.

	Total (N = 134)	Mild-to-moderate hearing loss (n = 69)	Severe-to-profound hearing loss (n = 65)	*p*
General demographic data				
Age (years)	52.2 ± 11.9	50.8 ± 11.4	53.7 ± 12.4	0.170
Sex, male/female	69/65	37/32	32/33	0.730
Vascular risk factors				
Hypertension	34 (25.4%)	15 (21.7%)	19 (29.2%)	0.330
Diabetes mellitus	22 (16.4%)	9 (13.0%)	13 (20.0%)	0.352
Cardiovascular disease	10 (7.5%)	4 (5.8%)	6 (9.2%)	0.523
Clinical characteristics				
Period to initial visit (days)	2.5 ± 2.1	2.7 ± 2.0	2.3 ± 2.2	0.304
Affected side, right/left	77/57	37/32	40/25	0.354
Initial PTA (dB)	72.0 ± 25.1	52.2 ± 12.8	92.9 ± 16.3	<0.001
Final PTA (dB)	42.4 ± 30.3	28.5 ± 19.4	57.2 ± 32.9	<0.001
Vertigo	28 (20.9%)	8 (11.6%)	20 (30.8%)	0.010

### Radiological characteristics of the vertebrobasilar system

The overall radiological assessment demonstrated that 71.6% (96/134) of the patients had a tortuous BA and 67.1% (90/134) had an asymmetric VA (Table 2). The mean diameter of the BA was 3.36 ± 0.54 mm. Only five patients showed an ectatic BA with a diameter larger than 4.5 mm.^12^ A dominant VA on the left side and BA displacement to the right side were more frequent. When the study populations were divided into two groups according to the degree of hearing impairment, there was no statistically significant difference between the groups in terms of BA diameter, BA tortuosity (side and degree), and VA asymmetry (Table 2).

**Table 2:** Radiological characteristics of the vertebrobasilar system.

	Total (N = 134)	Mild-to-moderate hearing loss (n = 69)	Severe-to-profound hearing loss (n = 65)	*p*
BA diameter (mm)	3.36 ± 0.54	3.31 ± 0.52	3.43 ± 0.55	0.305
BA tortuosity	96 (71.6%)	48 (69.6%)	48 (73.8%)	0.702
Side of BA displacement				0.883
Right	55 (41.0%)	27 (39.1%)	28 (43.1%)	
Left	41 (30.6%)	21 (30.4%)	20 (30.8%)	
Degree of BA laterality				0.854
Grade 0	38 (28.4%)	21 (30.4%)	17 (26.2%)	
Grade 1	37 (27.6%)	16 (23.2%)	21 (32.3%)	
Grade 2	39 (29.1%)	23 (47.1%)	16 (24.6%)	
Grade 3	20 (14.9%)	9 (13.0%)	11 (16.9%)	
VA asymmetry	90 (67.1%)	45 (65.2%)	45 (69.2%)	0.621
Side of dominant VA				0.594
Right	35 (26.1%)	17 (24.6%)	18 (27.7%)	
Left	55 (41.0%)	28 (40.6%)	27 (41.5%)	
Symmetric	44 (32.8%)	24 (34.8%)	20 (30.8%)	

### Relationships between radiological and audiological findings

**Figure 2:**
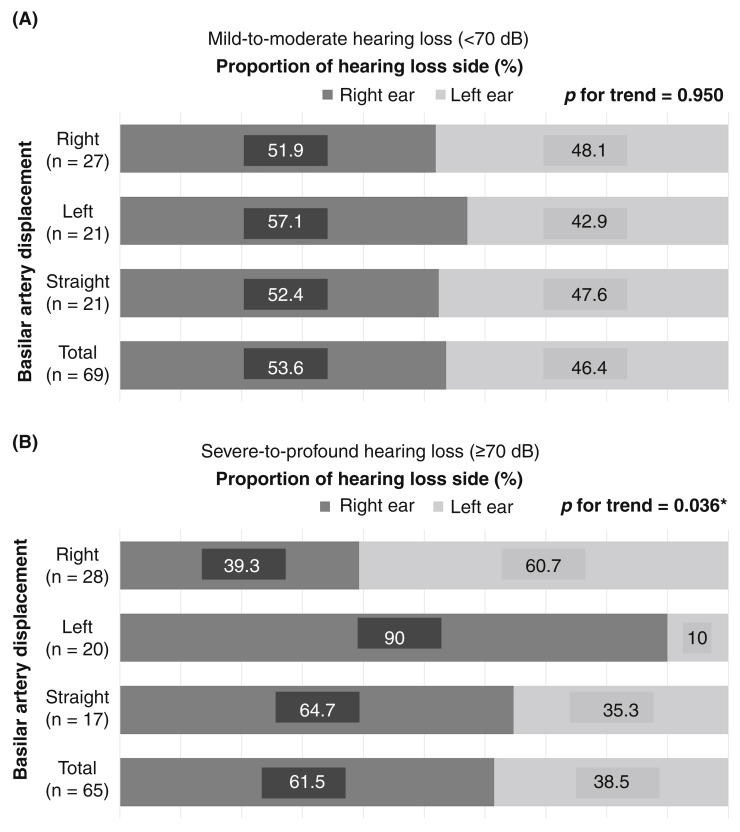
The relationship between the side of hearing loss and the side of basilar artery displacement for two groups classified according to the degree of hearing loss. A, Mild-to-moderate hearing loss group (initial pure tone average < 70 dB). B, Severe-to-profound hearing loss group (initial pure tone average ≥ 70 dB).

**Figure 3:**
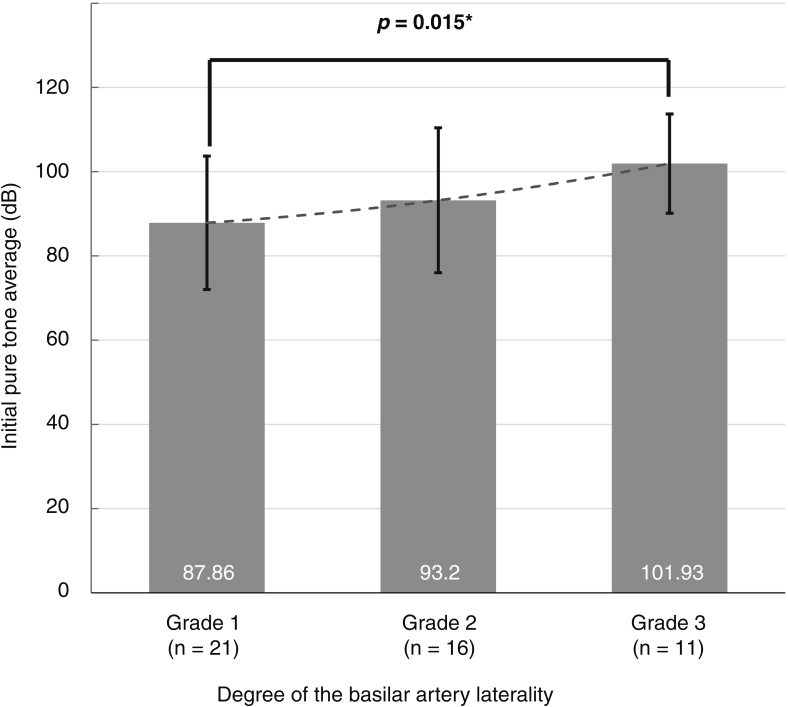
The degree of hearing impairment at the initial visit in the severe-to-profound hearing loss group. Subgroup analysis according to the degree of basilar artery laterality.

In this study, right-sided hearing loss (identified in 57.5% of patients) and right-sided BA displacement (identified in 41.0%) were more frequently seen than left-sided hearing loss and displacement. However, there was no significant directional relationship between the sides of hearing loss and BA displacement (*p* =  0.152, linear-by-linear association). We further analyzed the directional relationship within each hearing level group (PTA < 70 dB, and PTA ≥ 70 dB). Fig. 2 shows the significant difference in the trends between the two groups. Hearing loss laterality in the mild-to-moderate hearing loss group was not associated with the side of BA displacement (*p*  = 0.950). On the other hand, in the severe-to-profound hearing loss group, 48 (73.8%) patients had lateral displacement of the BA (≥ Grade 1), and a significant directional relationship was detected, with hearing loss more likely to be on the opposite side to BA displacement (*p* =  0.036). Additionally, the degree of hearing impairment in the severe-to-profound hearing loss subgroup of patients with a tortuous BA was associated with the degree of BA tortuosity (Fig. 3). Our subgroup analysis showed that the initial PTA was significantly higher in patents with a Grade 3 BA displacement than patients with a Grade 1 BA displacement (*p* =  0.015).

### Hearing recovery

**Figure 4:**
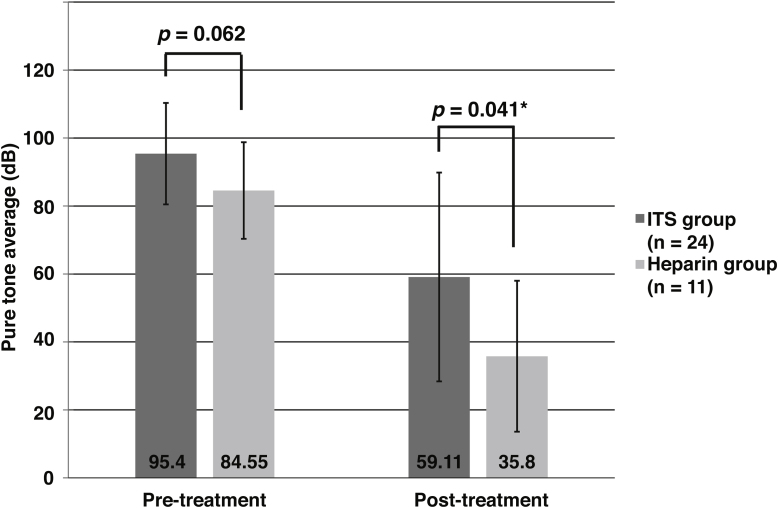
Assessment of hearing level at the initial visit and at the three-month follow-up in selected patients with idiopathic sudden sensorineural hearing loss: 1) those with an initial pure tone average ≥ 70 dB, and 2) those with displacement of the basilar artery to the contralateral side. Comparison of the therapeutic effects of heparin and intratympanic steroid injection as adjuvant treatments for these patients.

The above results indicate that our hypothesis on vascular etiology was valid only in the severe-to-profound hearing loss group. Thus, further analyses of the efficacy of adjuvant therapies were performed in patients with initial PTA ≥ 70 dB, all of whom received adjuvant therapy. The frequency of vertigo was 36.2% in patients treated with ITS (ITS group, n = 47) vs. 16.7% in patients treated with heparin (heparin group, n = 18) (*p*  = 0.108). With regard to the initial PTA, the ITS group had poorer hearing than the heparin group (*p*  = 0.015) (Table 3). However, final hearing outcomes, absolute hearing gains, and hearing recovery did not significantly differ between the two groups (Table 3). Among 16 patients with no improvements, 9 patients (8.0% of the ITS group vs. 4.3% of the heparin group, *p* =  0.108) showed worsening of hearing from initial hearing level, which was between 1.25–12.5 dB worse. To select cases that were more likely to experience a hearing improvement following heparin therapy, we hypothesized that anticoagulant therapy may be more efficient in the subgroup of patients who had ISSNHL of vascular origin. Among the 65 patients in the severe-to-profound hearing loss group, 35 patients had hearing loss on the contralateral side of BA displacement. Our analysis of this subgroup showed that these patients achieved greater benefit from heparin than ITS as an adjuvant therapy (Fig. 4). In the heparin group, the follow-up PTA at three months improved to 35.8 ± 22.2 dB compared with 59.1 ± 30.7 dB in the ITS group (*p* =  0.041).

**Table 3:** Comparison of hearing measures in patients with severe-to-profound hearing loss.

	Total (n = 65)	ITS group (n = 47)	Heparin group (n = 18)	*p*
Initial PTA (dB)	92.9 ± 16.3	96.0 ± 16.2	85.0 ± 14.2	0.015
Final PTA (dB)	57.3 ± 32.9	62.1 ± 32.6	44.7 ± 30.8	0.076
Hearing gain (dB)	35.7 ± 23.2	33.9 ± 22.8	40.3 ± 24.4	0.321
Hearing recovery outcomes based on Siegel’s criteria				0.072
Complete recovery	13 (20.0%)	6 (12.8%)	7 (38.9%)	
Partial recovery	12 (18.5%)	9 (19.1%)	3 (16.7%)	
Slight improvement	24 (36.9%)	20 (42.6%)	4 (22.2%)	
No improvement	16 (24.6%)	12 (25.5%)	4 (22.2%)

There were no significant complications during or after heparin therapy, including hemorrhage, hypersensitivity, thrombocytopenia, or priapism. In addition, no patient experienced serious adverse events related to ITS, including tympanic membrane perforation, otitis media, and vertigo.

## Discussion

Interruption of the vascular supply and resultant cochlear ischemia is thought to contribute to ISSNHL in some cases. In the context of this vascular etiology, heparin adjuvant therapy to improve cochlear blood flow has been implemented for the treatment of some ISSNHL cases in our department. Since treatment decisions were generally based on the clinician’s preference rather than on findings that implied an underlying etiology, this intervention improved hearing in some patients but not others. Therefore, this study was designed to identify possible vascular causes for ISSNHL using MRI of the vertebrobasilar system and to help determine which patients will benefit from heparin adjuvant therapy. It is of particular clinical importance to note that both a severe-to-profound degree of initial hearing impairment (PTA ≥ 70 dB) and the presence of hearing loss on the opposite side to that of BA displacement were associated with an increased chance of success of heparin therapy.

The present study is the first to use MRI to evaluate the benefit profile of anticoagulant adjuvant therapy in patients with unilateral ISSNHL. The current analyses provide evidence that anticoagulant therapy was associated with a better hearing outcome in the subset of patients for whom a vascular etiology was inferred from MRI findings regarding the vertebrobasilar system. A strength of this study is that routine evaluation for retrocochlear pathology was performed using temporal bone MRI for cases of sudden sensorineural hearing loss. Considering the significant prevalence (4.4%−13.75%) of pathogenic MRI abnormalities, including vestibular schwannoma and pontine infarction, in patients with sudden sensorineural hearing loss,^9^ exclusion of these abnormalities through MRI supports an idiopathic diagnosis and reduces the risk of bias from diagnostic heterogeneity. In addition, the temporal bone MRI protocol in our department routinely included high resolution T2-weighted three-dimensional sequences. The cisternographic effect of these sequences enables the reliable identification of vascular structures within the cerebrospinal fluid such as the vertebral artery, the basilar artery, and even the Anterior Inferior Cerebellar Artery (AICA) without the risks of using contrast medium (Fig. 1B).

Some previous studies have reviewed the relationship between ISSNHL and the vertebrobasilar system. In 2016, Kim et al. conducted a study of 121 patients to analyze the characteristics of the vertebrobasilar system in relation to ISSNHL laterality.^6^ In accordance with the present study, they found that an opposite direction of BA displacement was significantly associated with ISSNHL laterality. In contrast to our findings, left-sided hearing loss (57.9%) was predominant in their study, and they did not report the opposite directional relationship for right-sided hearing loss. Other studies reviewed the relationship between ISSNHL and the characteristics of the AICA, which is a branch of the BA.^19^, ^20^ However, these authors found no significant correlations between ISSNHL and the anatomical shape and location of the AICA.

Currently, it is impossible to directly detect cochlear ischemia using a noninvasive method. Thus, it is difficult to precisely determine a vascular etiology of ISSNHL without pathological confirmation. On the other hand, the acute onset of sensorineural hearing loss in patients with vertebrobasilar ischemic stroke is commonly expected to result from a vascular cause, and this diagnosis is easily made using MRI.^21^ A recent study examining a consecutive case series of unilateral pontine infarction in the AICA territory showed a similar directional association with BA displacement as in the current study. Hong et al. reported that pontine infarcts on the opposite side to the direction of BA displacement occurred in 72.3% (34/47) of patients.^22^ Given these findings, our results could substantiate the previous hypothesis that atherothrombotic events may occur more frequently on the opposite side to that of BA displacement, and these can eventually lead to ISSNHL of a vascular etiology.^5^

Interestingly, the initial degree of hearing impairment had a significant influence on the directional relationship between hearing loss laterality and the direction of BA displacement. The group of patients who had mild-to-moderate hearing loss (PTA < 70 dB) at initial presentation did not show a relationship between the laterality of hearing loss and the side of BA displacement (Fig. 2A). Common variations in anastomoses between the AICA and collateral arteries could explain this finding.^23^ Experiments in guinea pig models have shown that the Posterior Inferior Cerebellar Artery (PICA) plays an important role in cochlear blood supply when the AICA or other branches of the BA are occluded.^24^ A cadaveric study has reported that anastomosis between the AICA and the PICA was observed in 27% of temporal bones.^25^ This suggests that if atherothrombotic events occur in the BA or its branches, severe hearing impairment can easily follow as a result of cochlear ischemia in cases without collateral cochlear arteries. On the contrary, in cases with collateral arteries to the cochlea, hemodynamic changes that occur in the displaced BA may not be enough to affect cochlear circulation and cause severe hearing loss. Unfortunately, none of our patients underwent conventional angiography, and confirming the existence of anastomosing vessels from the PICA is beyond the resolution of MRI.

The contribution of BA tortuosity to cochlear ischemia can be explained by the effect of traction of BA branches, as well as a procoagulant state associated with slow blood flow and reduced shear stress on the concave side of the vessel.^5^ Therefore, when patients with BA tortuosity develop ISSNHL in the contralateral ear, anticoagulant therapy is deemed to be a reasonable adjuvant treatment. However, in rare cases, labyrinthine hemorrhage may also be the vascular etiology of ISSNHL.^26^ Since it is known that systemic anticoagulation itself can increase the risk of labyrinthine hemorrhage, the use of heparin could constitute a potential harm to some patients with ISSNHL.^27^ Labyrinthine hemorrhage can be suspected by hyperintense signal in the T1-weighted sequence.^28^ In this study, only 1 of 134 (0.75%) patients had a hyperintense cochlear signal in T1. That patient was treated with ITS, resulting in improvement of hearing (27.5 dB). Moreover, the dose of heparin in our protocol is even lower than that in standard low-dose heparin regimens for thromboembolism prophylaxis.^29^ Based on our results, we believe that heparin adjuvant therapy has a role in selected patients with ISSNHL of obstructive vascular etiology. We have identified a combination of two independent factors that can be used to predict the likelihood of benefit from heparin adjuvant therapy, namely the presence of severe-to-profound hearing loss pretreatment (PTA ≥ 70 dB) and BA displacement to the contralateral side from the affected ear. However, heparin therapy for ISSNHL, in addition to being unproven in efficacy, is currently not recommended by any treatment guidelines and this study does not aim to validate its use in the clinical practice. Nevertheless, we anticipate that these two factors may predict the efficacy of other consensus recommendations targeting cochlear ischemia, such as hyperbaric oxygen therapy, in patients with ISSNHL.^30^

This study has some limitations. The main limitation are lack of randomization and sample size because of its retrospective nature. The analysis of the effectiveness of adjuvant therapy for treating patients with ISSNHL was inferior to that of high quality randomized clinical trials. However, it is believed that significant bias may be reduced by the selection of adjuvant therapy without knowing the disease status. Patients were allocated randomly to one of four physicians in our clinic, and every physician used only one protocol consistently during study period. Second, this study only relied on clinical characteristics, audiological measures, and MRI findings of the vertebrobasilar system to diagnose cochlear ischemia. Therefore, the suggested explanations for the contribution of BA tortuosity to ISSNHL remains speculative. To overcome these limitations, randomized controlled prospective study of clinical cases is needed to confirm the results of this study.

## Conclusion

In a subset of patients with severe-to-profound ISSNHL, BA tortuosity had a significant directional relationship with ISSNHL laterality. In these selected patients who meet specific conditions, a significantly positive therapeutic effect of heparin on improving the recovery of hearing was observed.

## Funding

This work was supported by the Gachon University Gil Medical Center (Grant number: FRD2019-01).

## Conflicts of interest

The author declares no conflicts of interest.
